# The protective role of phlorizin against lipopolysaccharide-induced acute orchitis in mice associated with changes in gut microbiota composition

**DOI:** 10.3389/fvets.2024.1340591

**Published:** 2024-05-23

**Authors:** Qing Guo, Tian-Feng Li, Jiang Huang, Jing-Chun Li, Ze-Cai Zhang, Yong-Li Qu

**Affiliations:** ^1^College of Animal Science and Technology, Heilongjiang Bayi Agricultural University, Daqing, Heilongjiang, China; ^2^Heilongjiang Key Laboratory of Efficient Utilization of Feed Resources and Nutrition Manipulation in Cold Region, Heilongjiang Bayi Agricultural University, Daqing, Heilongjiang, China

**Keywords:** phlorizin, orchitis, blood testosterone barrier, testosterone, gut microbiota

## Abstract

**Objective:**

Orchitis is a common reproductive disease of male animals, which has serious implications to human and animal reproduction. Additionally, phlorizin (PHN), a common polyphenol in apples and strawberries, has a variety of biological activities, including antioxidant, anti-inflammatory, anti-diabetic, and anti-aging activities. We aimed to determine the protective effects and potential mechanisms of PHN in lipopolysaccharide (LPS)-induced acute orchitis in mice.

**Method:**

After 21 days of PHN pretreatment, mice were injected with LPS to induce testicular inflammation, and then the changes of testicular tissue structure, expression of inflammatory factors, testosterone level, expression of testosterone-related genes, adhesion gene and protein expression were detected, and the structural changes in the intestinal flora after PHN treatment were further detected by 16SRNA.

**Result:**

Our results demonstrated that PHN treatment reduced LPS-induced testicular injury and body and testicular weight losses. The mRNA expression levels of pro-inflammatory cytokines-related genes and antioxidant enzyme activity were also decreased and elevated, respectively, by PHN administration; however, PHN treatment also reduced the LPS-induced decrease in testosterone levels in the testes. Additionally, further studies found that PHN increased the expression of marker proteins zonula occludens-1 (ZO-1) and occludin associated with the blood testosterone barrier compared with that in LPS treatment groups. To further examine the potential mechanisms of the protective effect of PHN on LPS-induced testicular injury, we compared the differences of gut microbiota compositions between the 100 mg/kg PHN treatment group and the control group using 16SRNA. Metagenomic analyses indicated that the abundances of *Bacteroidetes*, *Muribaculaceae*, *Lactobacillaceae*, *uncultured bacterium f Muribaculaceae*, and *Lactobacillus* in the PHN treatment group improved, while potential microbes that can induce intestinal diseases, including *Verrucomicrobia*, *Epsilonbacteraeota*, *Akkermansiaceae*, and *Akkermansia* decreased in the PHN treatment group.

**Conclusion:**

Our results indicate that PHN pretreatment might alleviate orchitis by altering the composition of gut microflora, which may provide a reference for reducing the occurrence of acute orchitis in male animals.

## 1 Introduction

Male animals reproductive tract inflammation and infections are closely related to infertility, among which orchitis is an important reproductive disease in male animals. Orchitis can reduce spermatogenesis and sperm quality, thereby causing serious damage to human and animal reproduction and serious economic damage to animal breeding (1, 2). Many factors contribute to orchitis in both humans and animals, including bacterial and viral infections and several diseases, including autoimmune diseases (3), cryptorchidism (4), and obesity (5); therefore, developing new methods for orchitis prevention and treatment is important.

Spermatogenic cells at different stages of development, Sertoli cells, and Leydig cells play the most important roles in testicular function. Leydig cells are in the interstitial compartments of the testes and are responsible for testosterone production, which is crucial for the normal development of male sex organs, spermatogenesis, and sperm maturation (6). Additionally, the blood-testis barrier (BTB) is important for normal spermatogenesis. The BTB in the testes, which consists of adjacent Sertoli cells near the basal membrane of the seminiferous tubules, peritubular tissue encircling seminiferous tubules, and interstitial capillary endothelium are crucial to maintaining the microenvironment necessary for testicular function (7). It can isolate spermatogenic cells at all developmental stages from the circulatory system, thereby preventing the diffusion of various endogenous and exogenous toxic chemicals in mammals (8, 9). The main structural component of the BTB is the Sertoli intercellular junction complex, which is formed by the coexistence of several proteins, including gap junctions (GJ), tight junctions (TJ), and basic ectoplasmic specializations (ES) (10). Years of research has shown that the pathological pathway of testicular inflammation predominantly includes inflammatory cytokine imbalance (11), testosterone synthesis disruption (12), oxidative stress (11), and BTB disruption (13), which leads to the apoptosis of spermatocytes and spermatids.

**Figure 1:**
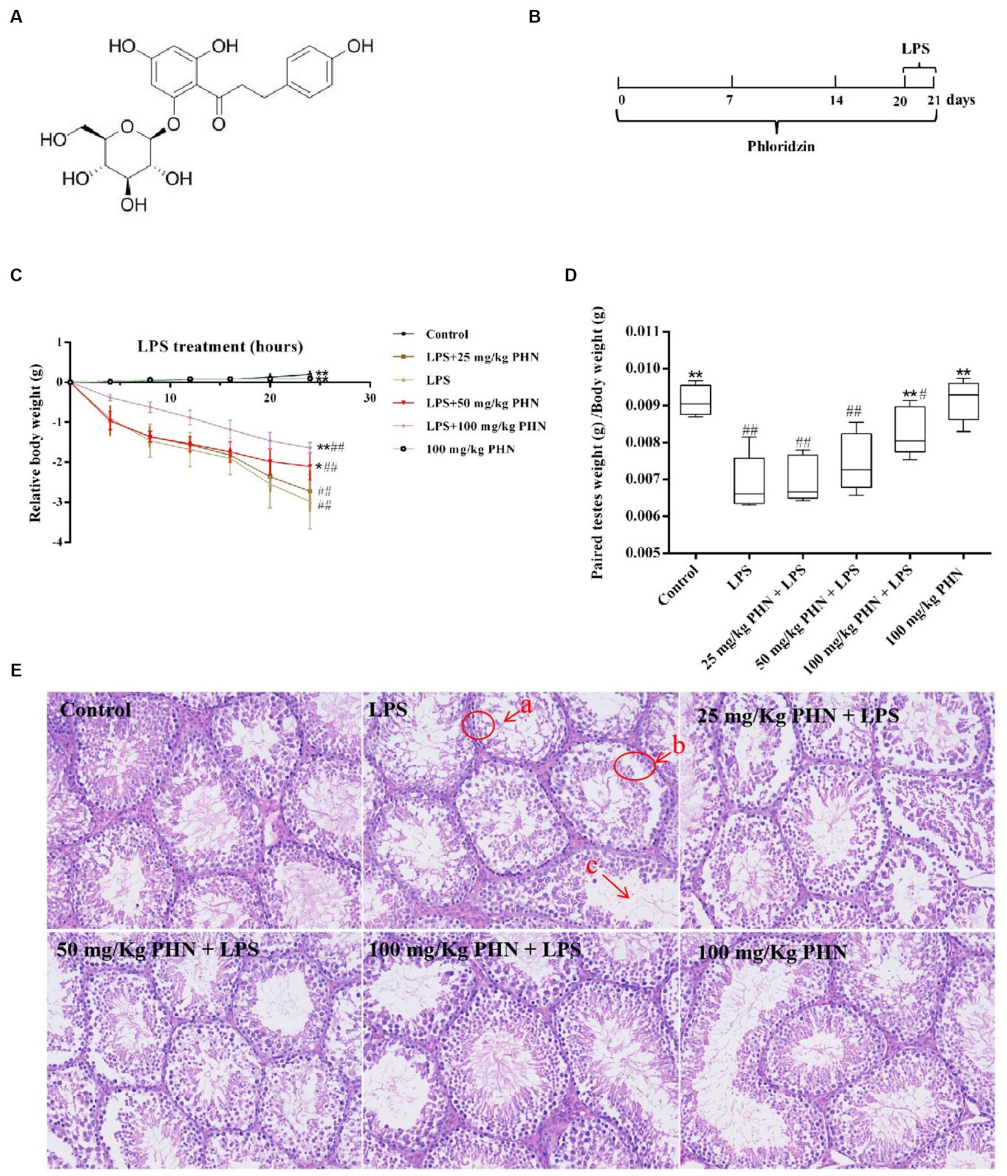
PHN decreased LPS-induced orchitis. **(A)** PHN chemical structure. **(B)** The experimental treatment protocol with PHN or LPS. **(C)** Relative changes in body weight. **(D)** Paired testes weight (g) /Body weight (g). **(E)** H&E staining, 200×. Data are demonstrated as means ± SD (*n* = 5). *p < 0.05 and **p < 0.01 vs. the LPS group; #*p* < 0.05 and ##*p* < 0.01 vs. the control group.

Phlorizin (PHN; Figure 1A) is a glucoside of phloetin, chemically named 1-[2-(beta.D-glucopyranosyloxy)-4,6-dihydroxyphenyl]-3-(4-hydroxyphenyl)-1-propanone, that belongs to the dihydrochalcone family of flavonoids (14, 15). It is found predominantly in the root bark, stem, young leaves, and fruits of apple trees (16, 17). PHN has many important biological activities, including antioxidant activity (18), blood sugar regulation (19), memory improvement (20), and anti-allergy (21) and anti-cancer activities (22) and has potential application value in the health product industry (23). Additionally, studies on obese mice induced by high-fat diets showed that dietary PHN can ameliorate the redox state, and its main mechanism is closely related to gut microbiota variations (24). PHN can also reduce blood lipopolysaccharide (LPS) level and increase insulin sensitivity in obese mice and mice with type 2 diabetes by regulating gut microbiota variations (25); however, orchitis can be induced by bacterial LPS, which can result in failure of spermatogenesis and damage to the BTB (26, 27). Therefore, we used an LPS-induced acute orchitis mouse model to study whether PHN can prevent acute orchitis and to explore its potential mechanisms.

## 2 Methods

### 2.1 Chemicals

PHN (>98% HPLC) was purchased from Chengdu Preferred Biotechnology Co., Ltd. (Chengdu, China). Glutathione (GSH), superoxide dismutase (SOD), and malondialdehyde (MDA) kits were purchased from Nanjing Jiancheng Bioengineering Institute (Nanjing, China). All other chemicals used in this study were of analytical reagent grade. Unless otherwise stated, all chemicals used in this study were purchased from Sigma Chemical (St Louis, MO, United States).

### 2.2 Animals

Male C57BL/6 (*n* = 72) mice with similar body weight (21–23 g) were purchased from the Laboratory Animal Department of the Harbin Medical University (Harbin, China) and housed at 24 ± 1°C and received food and water *ad libitum*. The mice were housed in a clean environment to strict ensure animal welfare.

### 2.3 Testitis induction and evaluations

In this study, LPS (5 mg/kg) was intraperitoneally injected to induce acute orchitis in mice (11). Mice were randomly divided into six equal groups, that is, the control, LPS, PHN (25, 50, and 100 mg/kg) with LPS, and PHN (100 mg/kg) groups. Phloridzin was dissolved in distilled water and orally administered to animals at dosages of 25, 50 and 100 mg/kg body weight daily throughout the experimental period. The experimental timelines for the animal models are shown in Figure 1B. In the PHN (25, 50, and 100 mg/kg) + LPS groups, mice were intragastrically administered their respective PHN dose 21 d before LPS treatment daily. Mouse orchitis was induced by intraperitoneal LPS injection at 5 mg/kg (PHN weight/mouse body weight) for 24 h (from day 20 to 21). Twenty-four hours after injection of LPS, mice were euthanized and testes were collected to detect tissue structure, expression of factors associated with inflammation and testosterone synthesis, and integrity of the blood-testosterone barrier. In addition, blood is collected and then centrifuged to obtain serum, which is assayed for testosterone content. Moreover, mice in the PHN groups were continuously treated with their respective PHN doses during LPS treatment.

### 2.4 Oxidative stress and myeloperoxidase assay

Glutathione (GSH), superoxide dismutase (SOD), malondialdehyde (MDA), and myeloperoxidase (MPO) activity in the teste tissues from different groups was examined using their corresponding kits (Nanjing Jiancheng Bioengineering Institute) in accordance with the manufacturer protocols. The enzymatic activity was measured by a microplate reader (Bio-Rad, United States of America) according to the respective absorbance.

### 2.5 Testosterone assay by ELISA tests

ELISA kits (Nanjing Jiancheng Bioengineering Institute, Nanjing, China) were used to measure testosterone levels in the serum samples. Approximately 500 μL of blood samples were obtained from each mouse, and serum samples were collected by centrifugation at 1000 g for 12 min at 4°C. Serum testosterone levels were measured using kits according to the manufacturer protocols by a microplate reader (Bio-Rad, United States of America).

### 2.6 Quantitative real-time polymerase chain reaction

Total RNA was isolated from teste samples from different treatment groups using TRIzol reagent (Invitrogen), according to the manufacturer instructions. The RNA concentration was measured using a NanoDrop 2000c spectrophotometer (Thermo Fisher Scientific), and complementary DNA (cDNA) was synthesized using a SuperScript III First-Strand Synthesis System (Invitrogen, Carlsbad, CA, United States). Real-time fluorescence quantitative PCR was performed using a CFX96 Touch Real-Time PCR Detection System (Bio-Rad, Hercules, CA, United States) and SYBR Green Plus reagent kit (TransGen Biotech, AQ141, Beijing, China). Primer sequences used in this study are listed in Table 1 with β-Actin was used as the reference gene. Quantitative RT-PCR was conducted thrice and normalized to the expression of the reference gene (i.e., β-actin). The relative gene expression levels were calculated using the 2^-ΔΔCT^ method.

**Table 1:** Oligonucleotide primers used for qRT-PCR.

Gene name	Primer sequence (5′-3′)
TNF-α	F:AATTACCTCAGGCAGTGTCTCAGTTG R:CACCGTGTCCTTGTCAGCTTGG
IL-1β	F:TCGCAGCAGCACATCAACAAGAG R:AGGTCCACGGGAAAGACACAGG
IL-2	F:GAGCAGGATGGAGAATTACAGGAACC R:GCCGCAGAGGTCCAAGTTCATC
IL-17A	F:ACGTTTCTCAGCAAACTTAC R: CCCCTTTACACCTTCTTTTC
stARF	F:TCTCTAGTGTCTCCCACTGCATAGC R:TTAGCATCCCCTGTTCGTAGCT
3β-HSD	F: CAAGTGTGCCAGCCTTCATCT R: TTCATGATTCTGTTCCTCGTGG
ZO-1	F:TTCTTGCAAAGTATCCCTTCTGT R:GAAATCGTGCTGATGTGCCA
Occludin	F: GTCCTCCTGGCTCAGTTGAA R: CGGACATGGCTGATGTCACT
β-Actin	F: TGCTGTCCCTGTATGCCTCT R: TGTCACGCACGATTTCCC

F, forward; R, reverse.

### 2.7 Hematoxylin–eosin staining

Teste tissues obtained from different treatment groups and were fixed in 4% (v/v) paraformaldehyde for 24 h, embedded in paraffin, and cut into 5 μm sagittal sections. The sections were de-paraffinized with xylene and ethanol, washed with phosphate-buffered saline (PBS), and permeabilized with 0.1 M citrate and 0.1% Triton X-100 permeabilization solution. The deparaffinized sections were stained with hematoxylin and eosin (H&E), and images were captured using an OLYMPUS BX53 microscope to examine pathological structural changes in the testes.

### 2.8 Immunofluorescence

Immunofluorescence staining was performed on paraffin-embedded sections of testicular tissue. Tissue slices were deparaffinized, rehydrated, and washed with 1% PBS-Tween. Sections were deparaffinized in xylene for 24 h and rehydrated. After washing with 1% PBS-Tween, the sections were treated with 3% hydrogen peroxide, permeabilized with 0.3% Triton X-100, and blocked with 3% BSA. Next, the sections were incubated for 1 h at 37°C with primary antibodies directed against Zona occludens 1 (ZO-1) or occludin (1:200; Beijing Biosynthesis Biotechnology Co., LTD, Beijing, China). After washing with PBS three times, the slides were incubated with species-specific fluorescent secondary antibodies (1:200; Beijing Biosynthesis Biotechnology Co., LTD, Beijing, China) for 1 h at 23°C and stained with Hoechst 33342. Finally, the cover slips were mounted, and images were captured using a light microscope (Olympus, Tokyo, Japan).

### 2.9 Gut microbiota analysis

Before the experiment began, each mouse was marked for subsequent experiments, and fresh fecal pellets were obtained after 20 d. Total DNA was extracted from the samples, primers were designed and synthesized in accordance with the conserved block, and the ends of the primers were connected with sequencing connectors to conduct PCR amplification. A sequencing library was established using product purification, quantification, and homogenization. The original data from high-throughput sequencing were analyzed and converted into sequence readings through base calling. The same operational taxonomic unit (OTU) was defined as a sequence greater than or equal to 97% for bacterial classification.

### 2.10 Statistical analysis

Statistical analysis of experimental data was performed using SPSS 17.0. The values are presented as mean ± standard deviation (SD), and multiple comparisons were analyzed using one-way ANOVA followed by Tukey’s multiple-comparison test. The differences between the two groups of data were assessed using an unpaired two-tailed Student t-test. Significant differences are represented by *p* < 0.05 and *p* < 0.01.

## 3 Results

### 3.1 Phlorizin alleviated LPS-induced orchitis

Excessive LPS in the body can cause systemic sepsis in animals, which can cause serious weight loss over a short period; therefore, we measured body weight variations of the different treatment groups every 4 h after LPS injection, and our results showed a significant decrease in the LPS treatment group compared to that in the control group; however, those treated with PHN (50 and 100 mg/kg) significantly decreased the LPS-induced weight loss (Figure 1C). Additionally, testicular weight loss is also an important marker of orchitis; therefore, we examined the relative changes in testicular and body weights. The results showed that LPS treatment significantly reduced testicular weight compared with the control group, while 100 mg/kg PHN treatment significantly restored this change (Figure 1D). Moreover, from histopathological observations, we found that the number of spermatogenic cells were decreased (Figures 1E-a), most spermatogenic cells were exfoliated (Figures 1E-b), and the mature sperm were rare (Figures 1E-c) in the LPS treatment group; however, PHN treatment partially reversed these changes in a dose-dependent manner.

### 3.2 Inhibition of MPO activity

**Figure 2:**
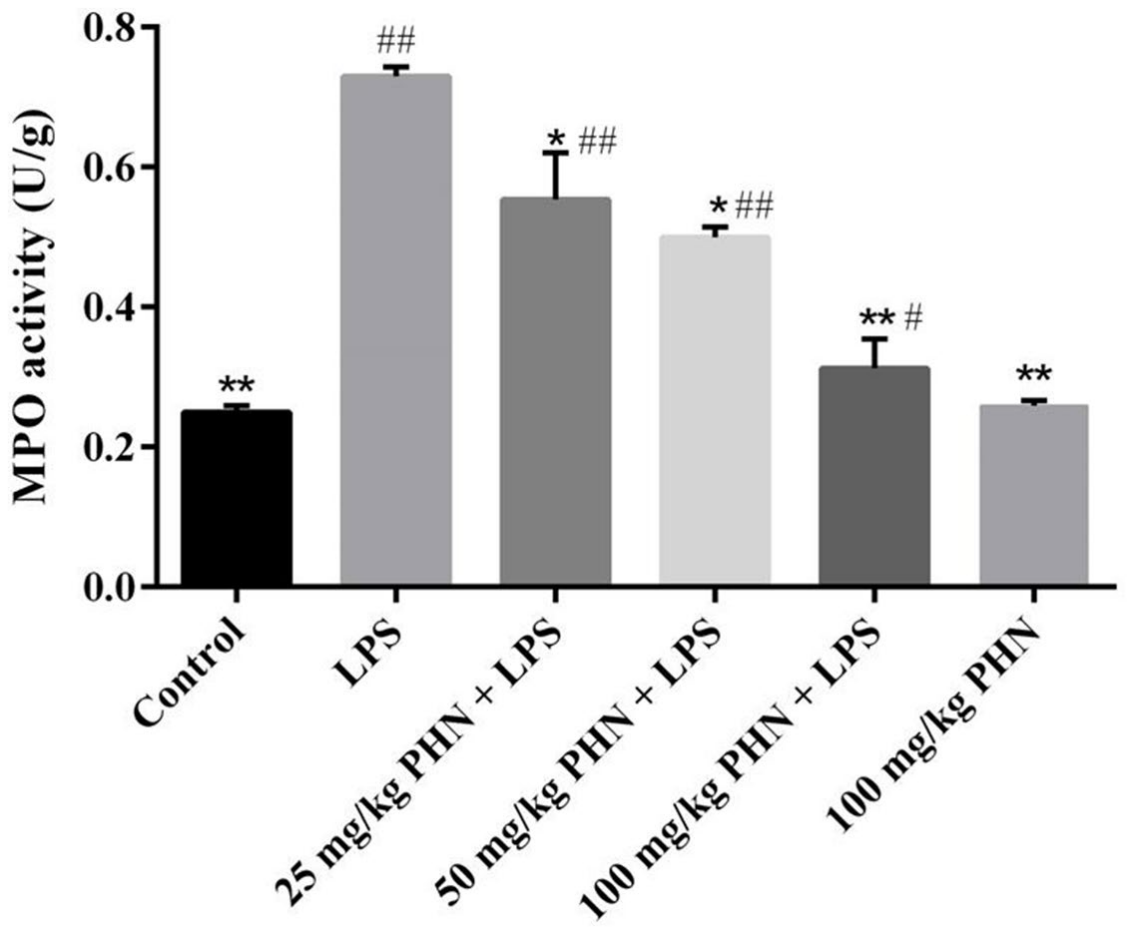
Decrease activity of MPO. Samples were collected from the testes homogenate of the experimental mice. Data are demonstrated as means ± SD (*n* = 5). *p < 0.05 and **p < 0.01 vs. the LPS group; #*p* < 0.05 and ##*p* < 0.01 vs. the control group.

Myeloperoxidase is abundant in neutrophils and is closely related to inflammation. Here, we examined the effect of PHN pretreatment on LPS-induced MPO activity in the testes using ELISA. As shown in Figure 2, LPS treatment increased a nearly three-fold MPO activity in the testes compared with the control group; however, compared with the LPS group, different concentrations of PHN significantly reduced MPO activity in a dose-dependent manner (Figure 2).

### 3.3 Inhibition of pro-inflammatory cytokines

**Figure 3:**
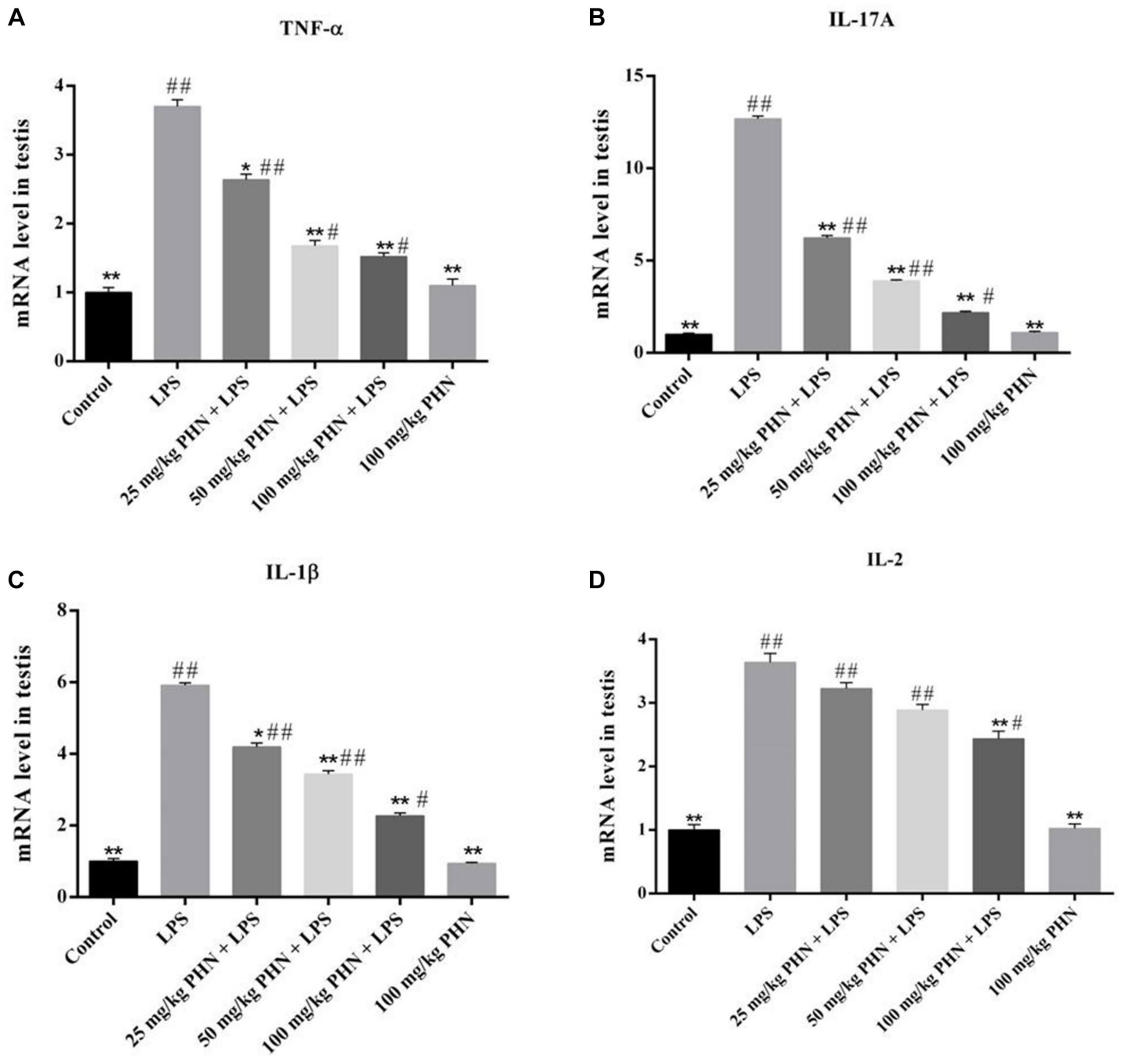
Reduction of pro-inflammatory cytokines. The mRNA expression levels of TNF-α **(A)**, IL-17A **(B)**, IL-1β **(C)** and IL-2 **(D)** in testes tissues were detected by qRT-PCR. Data are demonstrated as means ± SD (*n* = 5). *p < 0.05 and **p < 0.01 vs. the LPS group; #*p* < 0.05 and ##*p* < 0.01 vs. the control group.

Changes in pro-inflammatory cytokines damaged the Leydig cells and BTB, thereby resulting in decreased testosterone synthesis and spermatogenesis. Here, we examined the effect of PHN pre-treatment on LPS-induced inflammatory cytokine production in the testes. Our results indicated that LPS treatment significantly increased the mRNA expressions of Tumor necrosis factor α (TNF-α), Interleukin-17A (IL-17A),Interleukin-1β (IL-1β), and Interleukin-2 (IL-2) compared with those in the control (Figures 3A–D); however, different PHN concentrations significantly reduced the mRNA expressions of TNF-α, IL-17A, and IL-1β compared with those in the LPS treatment group (Figures 3A–C). Additionally, 25 mg/kg and 50 mg/kg PHN did not affect the mRNA expression of IL-2, while 100 mg/kg PHN significantly reduced the mRNA expression of IL-2 compared with that in the LPS treatment group (Figure 3D).

### 3.4 Improvement of oxidative stress

**Figure 4:**
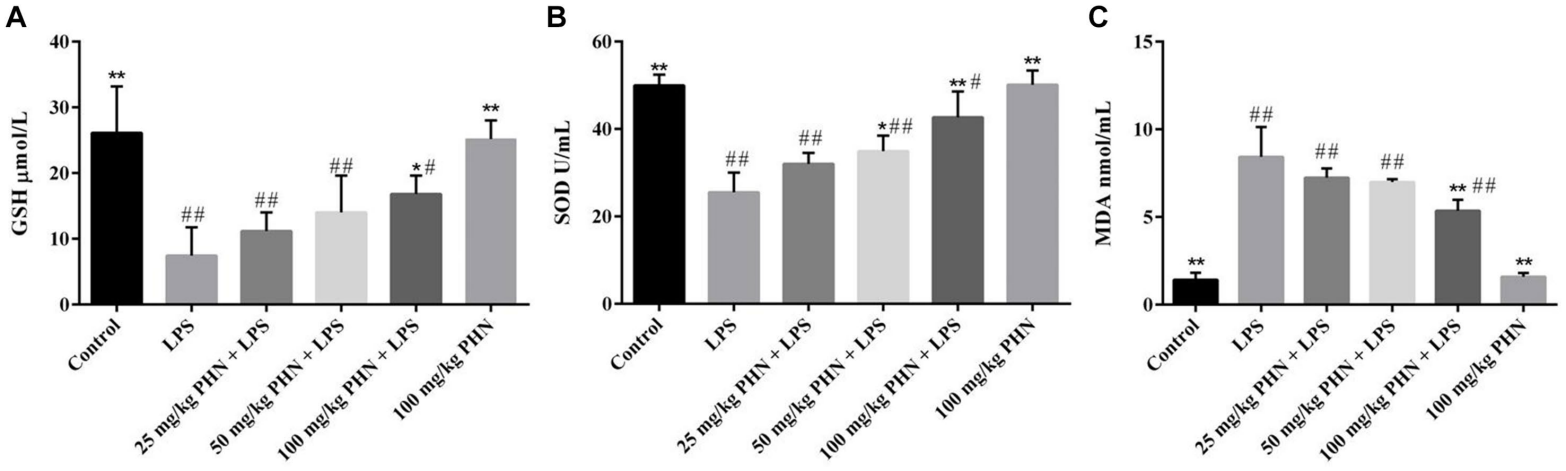
Amelioration of oxidative stress. **(A)** The activity of GSH. **(B)** The activity of SOD. **(C)**The production of MDA. Data are demonstrated as means ± SD (*n* = 5). *p < 0.05 and **p < 0.01 vs. the LPS group; #*p* < 0.05 and ##*p* < 0.01 vs. the control group.

To test whether PHN treatment has an antioxidant function in LPS-induced orchitis, we tested three oxidative stress markers. Our results showed that the expression levels of SOD (25.5 vs. 50.0, *p* = 0.002) and GSH (7.5 vs. 26.1, *p* = 0.006) in the LPS-induced acute orchitis group were significantly lower than those in the control group (Figures 4A,B). In contrast, the mice in the LPS group showed a significant increase in MDA (1.4 vs. 8.4, *p* = 0.015) concentration (Figure 4C). Compared with LPS group, after 50 and 100 mg/kg PHN treatment, the activity of SOD was significantly up-regulated (25.5 vs. 34.9, *p* = 0.049; 25.5 vs. 42.5, *p* = 0.017; Figure 4B). Moreover 100 mg/kg PHN treatment, GSH and MDA were significantly up-regulated (7.5 vs. 16.8, *p* = 0.042; Figure 4A) and down-regulated (8.4 vs. 5.3, *p* = 0.044; Figure 4C) compared with LPS group, respectively.

### 3.5 Enhancement testosterone levels

**Figure 5:**
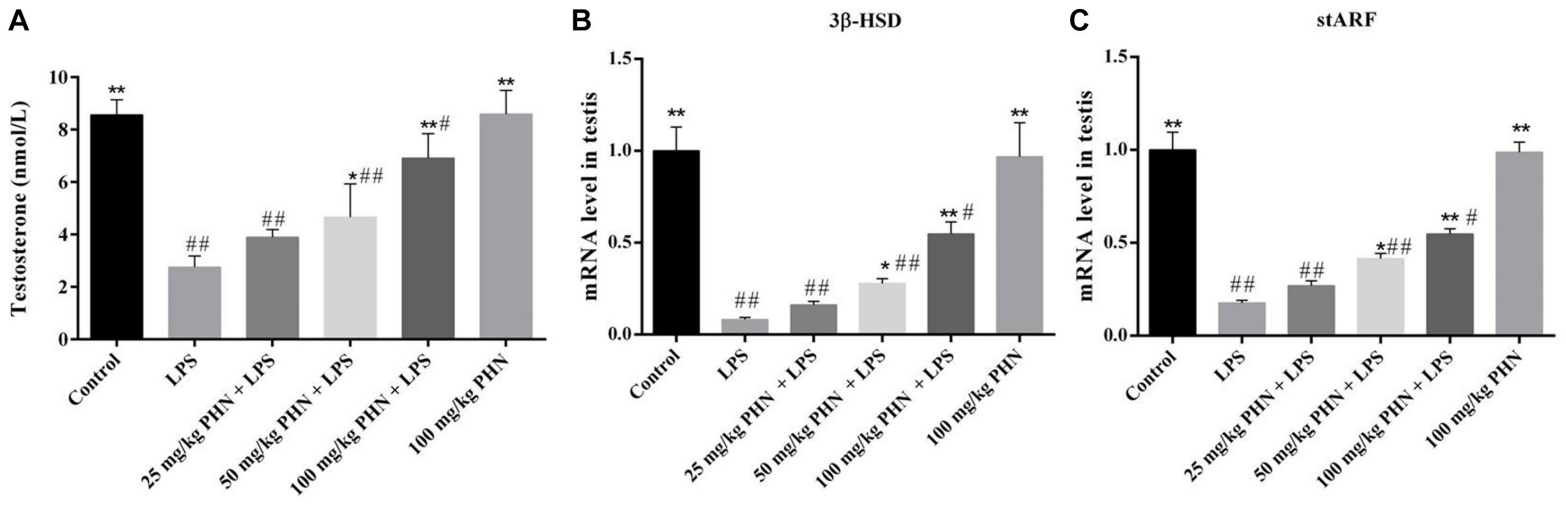
Improvement of testosterone production. **(A)** Testosterone levels in serum were detected by ELISA. The mRNA expression levels of 3β-HSD **(B)** and stARF **(C)** in testes tissues were detected by qRT-PCR. Data are demonstrated as means ± SD (*n* = 5). *p < 0.05 and **p < 0.01 vs. the LPS group; #*p* < 0.05 and ##*p* < 0.01 vs. the control group.

Testosterone plays an important role in spermatogenesis and maintenance of sperm motility in mammals; however, Leydig cells damaged during orchitis result in lower testosterone levels. As shown in Figure 5, our results indicate that the mRNA expression of genes associated with testosterone synthesis (3beta-hydroxysteroid dehydrogenase, 3β-HSD and steroidogenic acute regulatory protein, stARF) in the LPS group was significantly lower than that in the control group. Consistent with these results, the testosterone levels in the blood were also significantly decreased in the LPS group compared with that in the control group (2.8 vs. 8.6, *p* = 0.002; Figure 5A). However, these changes were significantly reversed after PHN treatment in a dose-dependent manner (2.8 vs. 3.9, *p* = 0.023; 2.8 vs. 4.7, *p* = 0.011; 2.8 vs. 6.9, *p* = 0.008; Figure 5A).

### 3.6 Enhance of the expression of ZO-1 and occludin

**Figure 6:**
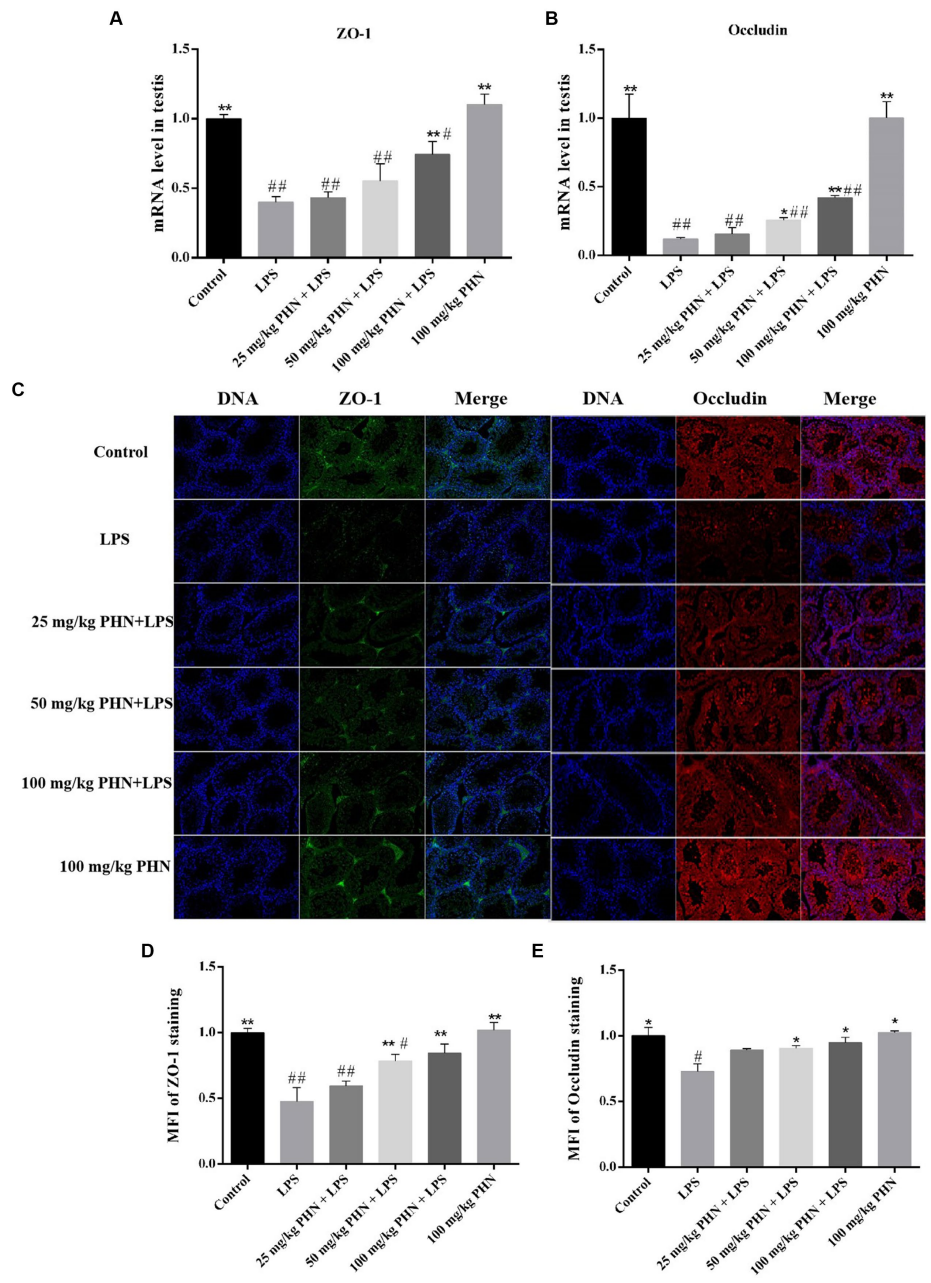
Enhance of the expression of ZO-1 and occludin. The mRNA expression of ZO-1 **(A)** and occludin **(B)** were measured by qRT-PCR in testes tissues. **(C)** The protein expression of ZO-1 and occludin were measured by immunofluorescence in testes tissues. 200×. Mean fluorescence intensity (MFI) of ZO-1 **(D)** and Occludin **(E)** staining as determined using Nikon NIS element software. Data are demonstrated as means ± SD (*n* = 5). *p < 0.05 and **p < 0.01 vs. the LPS group; #*p* < 0.05 and ##*p* < 0.01 vs. the control group.

The BTB is important in ensuring the microenvironment for independent spermatogenesis in the testes. Therefore, we examined the mRNA and protein expression of the BTB-associated tight junction proteins ZO-1 and occludin and found that the mRNA expression levels of ZO-1 and occludin in of the LPS-treated mice testes were significantly decreased (Figures 6A–B), and the protein content was also significantly decreased by immunofluorescence (Figures 6C–E). However, the mRNA and protein expression levels of ZO-1 and oocludin significantly recovered after PHN treatment in a dose-dependent manner (Figures 6C–E).

### 3.7 Effects of PHN treatment on gut microbiota at the phylum level

**Figure 7:**
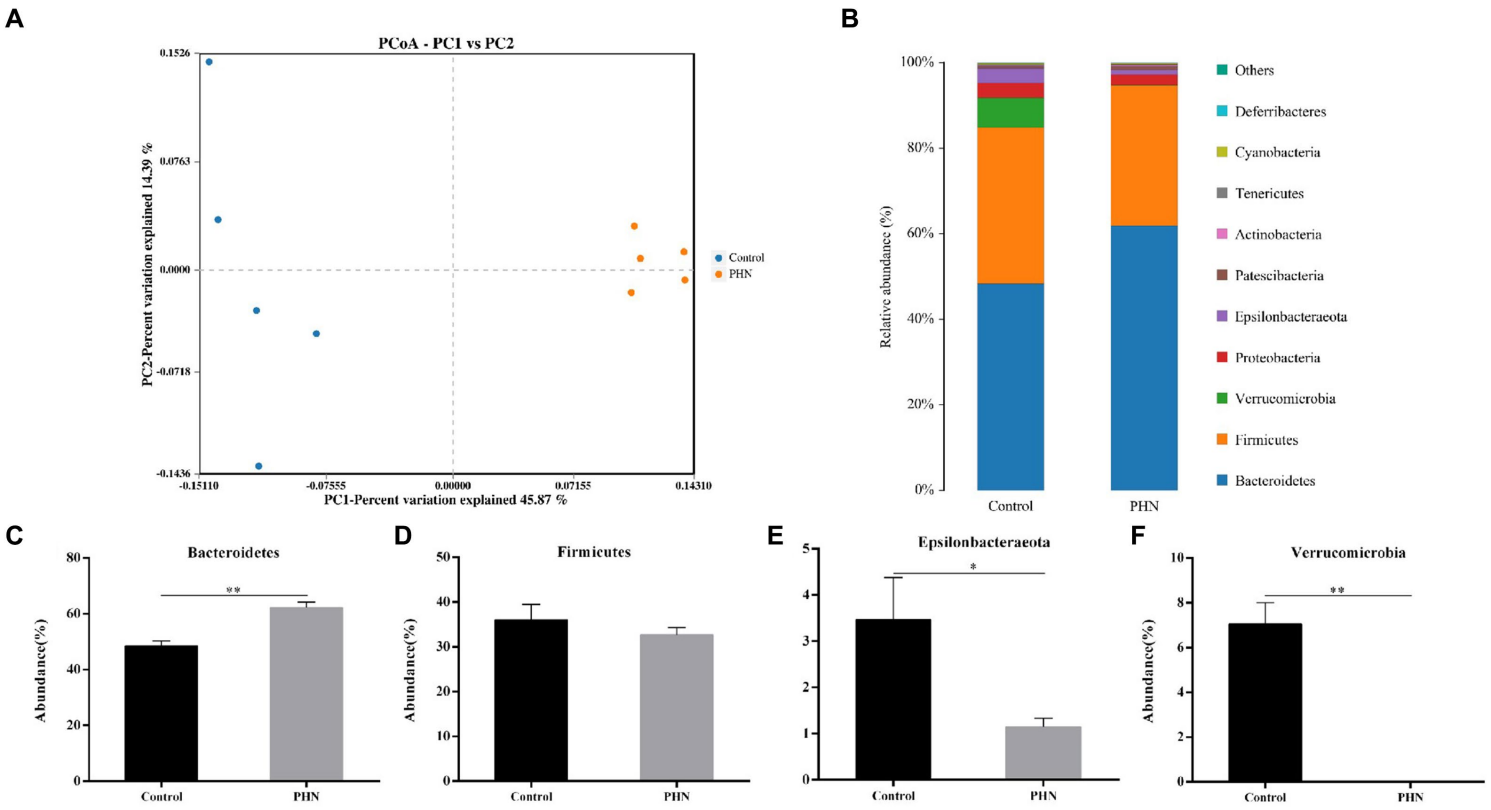
The relative abundance of gut microbiota after PHN treatment. **(A)** Beta diversity changes calculated by PCoA based on the OTU abundance. Each point represents the fecal microbiota of a mouse. **(B)** Relative abundance of the top 10 phyla were demonstrated as Bar graph. **(C–F)** Relative abundance of major phyla in the feces. Data are demonstrated as means ± SD (*n* = 5). *p < 0.05 and **p < 0.01 vs. the LPS group.

**Figure 8:**
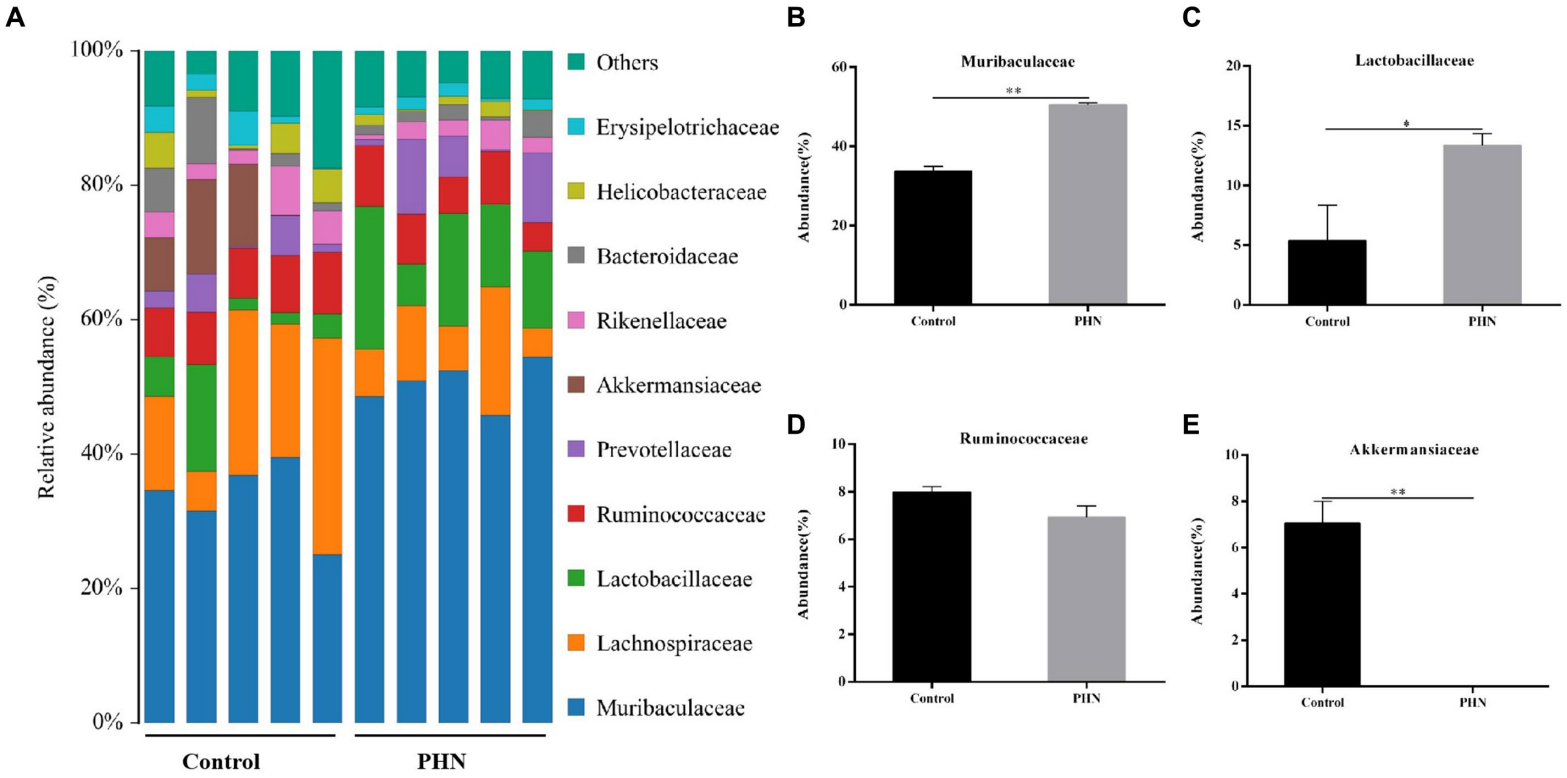
The relative abundance of gut microbiota at family level after PHN treatment. **(A)** Relative abundance of the top 10 family were demonstrated as Bar graph. **(B–E)** Relative abundance of major family in the feces. Data are demonstrated as means ± SD (*n* = 5). *p < 0.05 and **p < 0.01 vs. the LPS group.

The changes in microbial species, a clone library of the 16S rRNA gene, were established and sequenced to study the potential mechanism of PHN against LPS-induced orchitis in mice. For beta diversity analysis, principal coordinates analysis (PCoA) was used to analyze the microbiota communities. The data showed significant and distinct clustering of microbiota composition between the control and PHN treatment groups (Figure 7A), indicating that significant differences in microbial composition between the control and PHN treatment groups. Each sample contained 10 different phyla, including *Bacteroidetes, Firmicutes, Verrucomicrobia, Proteobacteria, Epsilonbacteraeota, Patescibacteria, Actinobacteria, Tenericutes, Cyanobacteria, and Deferribacteres* in descending order (Figure 7A). The four phyla with the highest abundances were *Bacteroidetes, Firmicutes*, *Verrucomicrobia*, *and Epsilonbacteraeota*. After PHN treatment, Firmicutes and proteobacteria did not change significantly (Figures 7C,F), but the *Bacteroidetes* abundance was significantly higher than that in the control group (Figure 7B). However, the ratio of Firmicutes to Bacteroidetes in the PHN-treated group was significantly lower than those in the control group (Figure 8D). Additionally, the *Epsilonbacteraeota* and *Verrucomicrobia* abundances in the PHN group were significantly lower than those in the control group (Figures 7E,G). Moreover, further analysis by LDA found that *Bacteroidetes* and *Verrucomicrobia* also showed significantly increase and decrease at phylum level (Supplementary Figure S1).

### 3.8 Effects of PHN treatment on intestinal microbiome abundance at the family and genus levels

**Figure 9:**
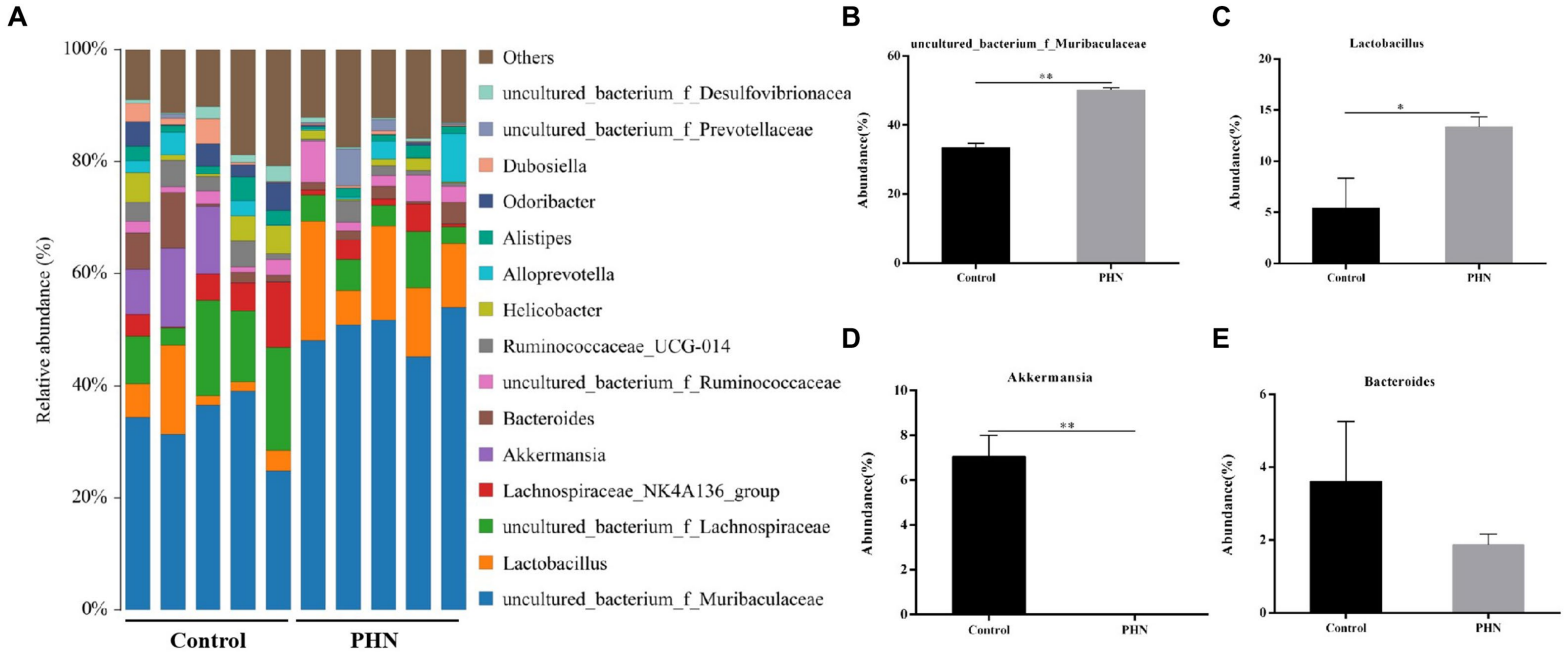
The relative abundance of gut microbiota at genus level after PHN treatment. **(A)** Relative abundance of the top 10 genus were demonstrated as Bar graph. **(B–E)** Relative abundance of major genus in the feces. Data are demonstrated as means ± SD (*n* = 5). *p < 0.05 and **p < 0.01 vs. the LPS group.

At the family level, *Muribaculaceae* was the most abundant in all fecal samples (Figure 8A). The proportion of *Muribaculaceae* in the PHN group was 50.52%, which was significantly higher than that in the control group (37.72%; Figure 8B). The second most abundant family in the PHN groups was *Lachnospiraceae* (9.20%),which was significantly lower than that in the control group (19.38%; Figure 8C). The third most abundant family in the PHN group was *Lactobacillaceae* (13.36%), which was significantly higher than that in the control group (5.40%; Figure 8D). The relative abundance of *Ruminococcaceae* (Figure 8E) did not change, but the relative Prevotellaceae and *Akkermansiaceae* abundances significantly increased and decreased (Figures 8F–G). At the genus level, *uncultured bacterium f Muribaculaceae* and *Lactobacillus* were significantly increased, and *Akkermansia* were significantly decreased in the PHN group compared with those of the control group (Figures 9A–E). Additionally, LDA analysis showed that similar results at the family and genus levels (Supplementary Figure S1).

## 4 Discussion

Orchitis is a complex infectious disease affecting the reproductive tract of male animals that has serious impacts on reproduction and reduces the quality of life. Damage to the BTB is difficult to repair; therefore, orchitis treatment is particularly difficult. PHN is a component of functional foods, primarily in apple peels, and has many biological activities, including antioxidative and anti-inflammatory activities; however, the preventive effect of PHN against LPS-induced orchitis in mice has not yet been reported. In this study, we examined the effects of different concentrations of PHN on orchitis and the underlying mechanisms. Similar to previous studies, body weight loss, testicular to body weight ratio decline, and testicular tissue structure damage were induced by LPS; however, we found that PHN significantly reduced LPS-induced changes as direct and indirect indicators of the severity of orchitis. These preliminary results suggest that PHN exerts a protective effect against LPS-induced orchitis.

LPS exposure of the testes promoted the secretion of TNF-α from Leydig cells (2). TNF-α is the earliest endogenous mediator of the inflammatory processes, while IL-17 is produced by highly differentiated Th17 cells and is an effective mediator of inflammation (28). Many studies have also shown that an imbalance between pro- and anti-inflammatory molecules, including TNF-α and IL-1β, in the testes can lead to orchitis (29, 30). In this study, the mRNA expressions of IL-2, TNF-α, IL-17A, and IL-1β, which have essential roles in inflammatory processes, were elevated in LPS-induced orchitis; however, PHN have been found to reduce their expression.

Testosterone is a steroid hormone secreted by Leydig cells that is important for sperm spermatogenesis and the maintenance of sperm motility (31). Inhibition of testosterone production disrupts spermatogenesis in humans and animals, leading to infertility in males (32). Additionally, Allen et al. demonstrated that LPS can damage mitochondria in Leydig cells by inducing an increase in Reactive Oxygen Species (ROS), inhibiting the synthesis and secretion of steroid hormones in Leydig cells, and reducing the expression of StAR and 3β-HSD genes (33). StAR-mediated cholesterol transport from the outer to inner mitochondrial membrane is a critical step during steroid formation (34–36). 3β-HSD is a steroid synthase that plays an important role in catalyzing the conversion of cholesterol steroid substrates to testosterone (37). Our study demonstrated that PHN could significantly restore LPS-induced decrease in testosterone content by increasing the mRNA expression levels of genes related to testosterone synthesis.

Furthermore, an increase in these pro-inflammatory cytokines causes an inflammatory response, which damages the structure of the BTB (2). The BTB divides the vas deferens into a basal compartment and lumen, which mainly includes basic ES, GJ, and TJ between Sertoli cells, providing a stable biochemical microenvironment for spermatogenesis (38, 39). Various TJ proteins, such as the ZO-1 complex, are involved in BTB formation. Moreover, another study reported that LPS elevation induced occludin downregulation and increased BTB permeability (27). Our study demonstrated that PHN reduced LPS-induced BTB damage in testes by increasing the expression of ZO-1 and occludin proteins.

The intestinal microbe is a large and diverse community of microbes, which is a substantial and complex ecosystem with mutual dependence on and restriction of the host. Therefore, the structure of the gut microbiota has an important impact on the health of the host. Recently, many studies have shown that gut microbiota is crucial to regulating male animal reproduction, including spermatogenesis and testosterone secretion (40, 41). Therefore, we examined whether PHN treatment could cause changes in the intestinal microflora structure, leading to reduced LPS sensitivity in mice. Previous studies have shown that PHN-treated mice showed no significant changes in *Bacteroidetes* and *Firmicutes* abundances at the phylum level in their feces (42); however, our study indicated that PHN treatment can lead to a significant increase in the relative abundance of Bacteroidetes. Short-chain fatty acids (SCFAs) concentrations were positively correlated with *Bacteroidetes* count (43). The phylum *Verrucomicrobia* is negatively correlated with obesity through the degradation of intestinal mucin (44). In this study, PHN treatment significantly increased and decreased the colonization of Bacteroides and Verrucomicrobia in the intestinal tract, which may be an important reason for PHN reducing LPS-induced orchitis in mice. *Epsilonbacteraeota* is harmful to the intestinal tract, which is significantly increased in dextran sulfate sodium (DSS)-induced colitis in mice (45). The relative abundance of *Epsilonbacteraeota* was significantly reduced, suggesting that PHN treatment could improve intestinal flora and thus enhance LPS resistance in mice. At the family level, *Muribaculaceae* is known as a short-chain fatty acid (SCFAs) producer with beneficial effects on intestinal homeostasis and health (46), and is also associated with the formation of the inner mucus layer in the colon and barrier function (47). Furthermore, propionate produced by *Muribaculaceae* plays an important role in the anti-inflammatory effects and maintenance of intestinal barrier function (47). In the present study, *Muribaculaceae* was important improve in PHN treatment compared with control group. Ruminococcaceae was also related to testicular function (48), but there was no significant change in its relative abundance. Furthermore, *Lactobacillaceae*, of the Firmicutes phylum, are well known for their role in digesting carbohydrates and their probiotic properties (49). The increased relative abundances of *Muribaculaceae* and *Lactobacillaceae* may be another important reason for the reduction in LPS-induced orchitis. Meanwhile, *Akkermansiaceae*, a group of mucin-degrading bacteria in the gut, decreased the integrity of the intestinal barrier function and led to increased permeability of the intestinal epithelium (50). In our study, the relative abundance of *Akkermansiaceae* decreased in PHN treatment than control group. These findings provide evidence that PHN treatment may enhance intestinal epithelial integrity, resulting in a decrease of LPS in the blood. The genus level data showed that PHN treatment significantly increased *uncultured bacterium f Muribaculaceae* and *Lactobacillus,* but decreased *Akkermansia. Uncultured bacterium f Muribaculaceae* could produce succinic acid, an important intermediate in the synthesis of propionic acid, through the degradation of polysaccharides (51). Furthermore, *Lactobacillus* is a beneficial bacterium in the intestinal tract that plays an important role in regulating health. Previous studies have shown that different types of *Lactobacillus* have anti-inflammatory properties (52), improve carbohydrate and fatty acid metabolism, and reduce lithocholic acid levels (53). Hao et al. reported that alginate oligosaccharide increased the sperm quality of mice with type 1 diabetes by increasing the *Lactobacillus* abundance (54). Similar to previous studies, PHN treatment significantly increased the abundance of *Lactobacillus* and *uncultured bacterium f Muribaculaceae*, which may explain PHN protection against LPS-induced testicular injury in mice. The relative abundances of *Akkermansia* were significantly lower in the PHN treatment group than in the control group. Akkermansia had anti-obesity effects, but a higher relative abundance of Akkermansia in the host intestinal tract could destroy intestinal mucins, which can promote colonic tumorigenesis and lead to intestinal inflammation (55, 56). These results indicate that PHN treatment significantly improved the intestinal microflora, which may be another important factor in the reduction of LPS-induced acute orchitis in mice.

In conclusion, our results indicate that PHN pretreatment might alleviate orchitis by altering the composition of gut microflora, which may provide a reference for reducing the incidence of acute orchitis.

## Data availability statement

The original contributions presented in the study are publicly available. This data can be found here: https://www.ncbi.nlm.nih.gov/bioproject/; PRJNA1105222.

## Ethics statement

The animal study was approved by Heilongjiang Bayi Agricultural University Committee of Ethics for Animal Welfare and Research. The study was conducted in accordance with the local legislation and institutional requirements.

## Author contributions

QG: Conceptualization, Funding acquisition, Methodology, Writing – original draft, Writing – review & editing. T-FL: Methodology, Writing – original draft. JH: Methodology, Writing – original draft. J-CL: Methodology, Writing – original draft. Z-CZ: Conceptualization, Funding acquisition, Methodology, Writing – review & editing. Y-LQ: Conceptualization, Funding acquisition, Writing – review & editing.
