# Supplementary figures and images for: The protective role of phlorizin against lipopolysaccharide-induced acute orchitis in mice associated with changes in gut microbiota composition
Source: Front Vet Sci. 2024 May 23;11:1340591. doi: 10.3389/fvets.2024.1340591 (PMC11156221; doi:10.3389/fvets.2024.1340591)

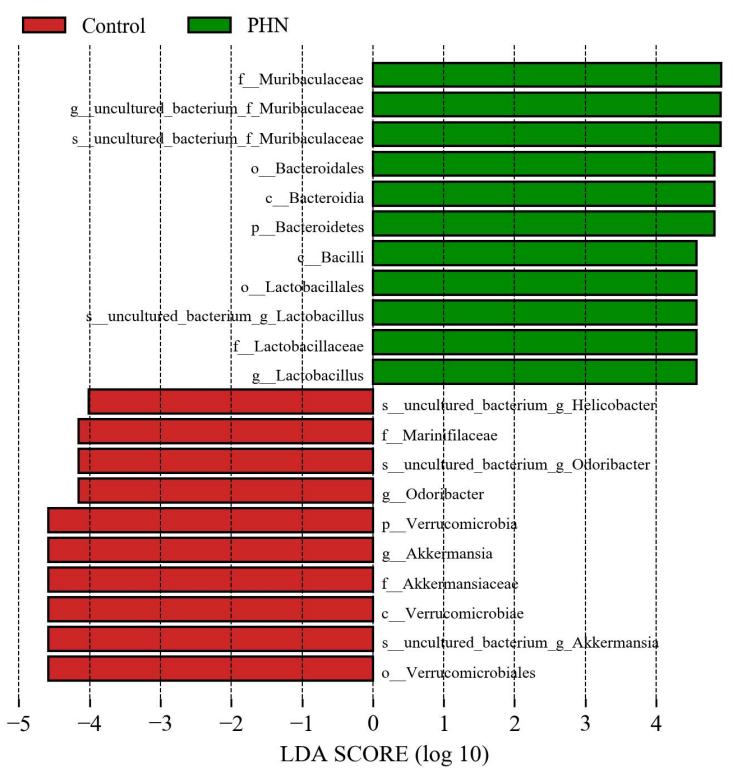

Supplement: Supplementary file 1 [file Image_1.JPEG]
